# Long-term remote sensing assessment of Natura 2000 protected areas in Poland (2004–2023)

**DOI:** 10.1038/s41598-026-42863-8

**Published:** 2026-03-07

**Authors:** Piejak Mateusz, Joanna Sender

**Affiliations:** 1 Department of Hydrobiology and Protection of Ecosystems, University of Life Sciences in Lublin, str. Dobrzańskiego 37, Lublin, 20-862 Poland; 2 Institute of Soil Science, Institute of Soil Science, Environment Engineering and Management, University of Life Sciences in Lublin, str. Leszczyńskiego 7, Lublin, 20-069 Poland

**Keywords:** Natura 2000, Remote sensing, Protected areas, Biodiversity monitoring, Land cover change, Climate resilience, Ecology, Ecology, Environmental sciences

## Abstract

Protected areas are essential for safeguarding biodiversity, yet their long-term environmental trajectories and stability remain insufficiently quantified at the national scale. This study evaluates two decades (2004–2023) of satellite-derived environmental dynamics within Poland’s Natura 2000 Special Areas of Conservation (SACs) using Landsat-based indicators: NDVI (vegetation greenness), TC Wetness (surface moisture), NDBSI (surface exposure related to land transformation), and LST (land surface temperature). Non-parametric trend analyses (Mann–Kendall and Sen’s slope) reveal that SACs maintained consistently high vegetation greenness with slower rates of increase than surrounding landscapes, consistent with relatively stable, long-established vegetation states. TC Wetness showed no consistent long-term directional trend, however, SACs exhibited slightly smaller declines during extreme drought periods, indicating greater persistence of moisture-related surface conditions. NDBSI declined more rapidly within SACs, suggesting lower levels of exposure surfaces commonly associated with anthropogenic land transformation, while LST remained statistically flat but consistently lower than in surrounding areas, indicating more stable thermal surface conditions. Taken together, these results indicate that areas currently designated as Natura 2000 sites follow more stable and internally consistent long-term environmental trajectories than the surrounding landscape, under shared climatic and land-use pressures. Our findings underline the importance of long-term remote sensing for assessing environmental trajectories and relative stability within protected area network.

**Supplementary Information:**

The online version contains supplementary material available at 10.1038/s41598-026-42863-8.

## Introduction

Protected areas are considered the cornerstone of biodiversity conservation, yet their effectiveness in maintaining long-term environmental conditions and resisting broad-scale pressures remains widely debated^1–3^. However, demonstrating conservation effectiveness at broad spatial and temporal scales remains challenging, particularly when long-term ecological change occurs under shared climatic and land-use pressures. In Europe, the Natura 2000 network is the world’s largest coordinated system of protected sites, covering more than 18% of EU land area^4,5^. It was established under the Habitats Directive (92/43/EEC) and the Birds Directive^6^/147/EC) as the main mechanism for implementing international biodiversity commitments, including the Convention on Biological Diversity (United Nations, 1992) and the Sustainable Development Goals of the 2030 Agenda^7^. However, methodological reviews highlight that remotely sensed assessments of protected area effectiveness still lack a unified framework^8^. While Natura 2000 was designed primarily to safeguard habitats and species, its potential contribution to broader environmental stability—such as moderating long-term land-use and climate-related pressures—remains insufficiently quantified at the national scale.

Most evaluations of Natura 2000 effectiveness rely on species- or habitat-level reporting, which, while essential, is limited by spatial gaps and irregular temporal coverage (Grodzińska-Jurczak & Cent, 2011). Landscape-scale assessments using consistent, long-term data are comparatively rare, despite their importance for understanding how protected areas function as ecological stabilizers under global change^9,4,10^. This knowledge gap is particularly relevant in Central and Eastern Europe, where land-use transformations, agricultural abandonment, and climate variability interact to reshape ecosystems^11–13^). As a result, relatively little is known about whether protected areas differ from surrounding landscapes in terms of long-term environmental trajectories and temporal consistency.

Remote sensing provides a powerful means of addressing this gap. Satellite-derived indicators allow consistent, multi-decadal monitoring of vegetation dynamics, hydrological conditions, land-cover change, and microclimatic processes^14,15,16,17^. By comparing such indicators between protected and non-protected landscapes, it becomes possible to examine whether protected areas exhibit more stable environmental trajectories under comparable disturbance regimes.

Poland hosts one of the largest national Natura 2000 networks in Central Europe, encompassing a wide range of forest, wetland, and grassland habitats. While several studies have analyzed regional trends in vegetation productivity or land-use change^18,19^, systematic assessments of long-term trajectories within Special Areas of Conservation (SACs) relative to national backgrounds remain scarce. This limits our ability to evaluate whether the network achieves its intended role of maintaining stable environmental conditions beyond species-level targets. Although designed to safeguard habitats and species, the network is highly heterogeneous, with human activities occurring at varying intensities across sites^20^.

The present study addresses this gap by conducting a 20-year analysis (2004–2023) of environmental indicators across Polish Natura 2000 SACs. Using Landsat-based time series, we evaluate four complementary indicators: NDVI (vegetation greenness), TC Wetness (surface moisture), NDBSI (bare soil and built-up exposure), and LST (land surface temperature). Specifically, we examine:

1. Do SACs exhibit distinct long-term trajectories compared to the national landscape?
2. To what extent do SACs exhibit more stable environmental dynamics under land-use change and climate variability?
3. What insights do these patterns provide for interpreting long-term environmental outcomes associated with large-scale conservation policy in Europe?

By addressing these questions, this study provides the first national-scale, multi-indicator assessment of Natura 2000 SACs in Poland over two decades, offering landscape-level evidence on long-term surface-level environmental trajectories associated with protected areas. At the same time, it demonstrates the utility of long-term remote sensing as a cost-effective approach for monitoring environmental patterns relevant to conservation policy evaluation. A key contribution of this study lies in distinguishing baseline environmental conditions from rates of long-term change across multiple surface-level indicators, providing a complementary perspective to studies focused solely on trend direction or absolute values.

## Materials and methods

### Study area

**Fig. 1:**
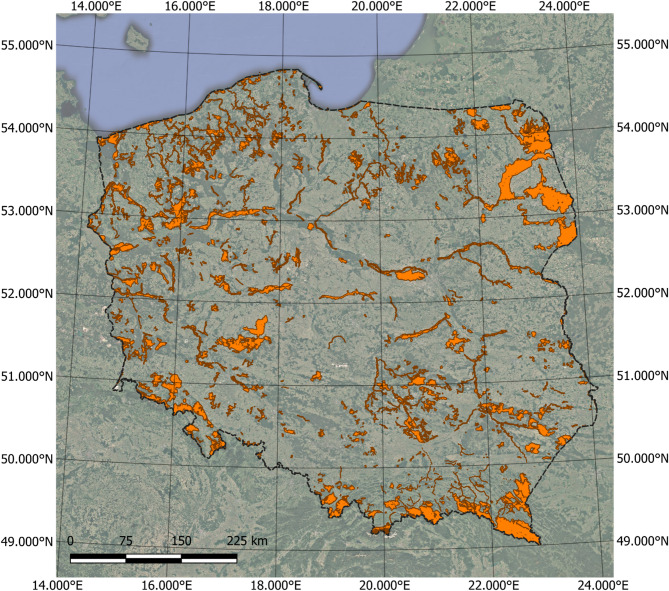
Spatial distribution of Natura 2000 SACs in Poland (sites < 250 ha were omitted).

The study covered the network of Natura 2000 Special Areas of Conservation (SACs) located in Poland. Only sites larger than 250 hectares were included in the analysis to minimize edge effects and mixed-pixel distortion inherent in moderate-resolution imagery (Fig. 1). Spatial boundaries were obtained from the General Directorate for Environmental Protection (GDOŚ), the national authority responsible for Natura 2000 management. After filtering, 330 SACs were retained, covering a total area of approximately 5.9 million hectares—representing about 18.8% of Poland’s territory. The remaining territory of Poland is used in this study as a national-scale background representing the broader environmental context rather than as a strictly comparable control area. Although Poland joined the European Union in 2004, the designation of Natura 2000 Special Areas of Conservation was not simultaneous across all sites. The majority of SACs were proposed and formally designated in the mid-2000s, while additional sites were designated or underwent boundary adjustments in subsequent years as part of the network’s gradual completion process. Therefore, the Natura 2000 network analyzed in this study represents a legally and administratively heterogeneous system with respect to the timing of designation.

Poland’s Natura 2000 sites span a wide range of ecosystems, including forests, wetlands, grasslands, and coastal zones, making them suitable for generalized national-scale assessments of long-term environmental dynamics. Many sites overlap with other forms of protection (e.g., nature reserves, national parks), but the Natura 2000 designation provides a common legal and management framework under the EU Habitats Directive (Council Directive 92/43/EEC). In this study, the analysis is conducted using the current official boundaries of Natura 2000 SACs, and the entire 2004–2023 time series is evaluated consistently for all sites. The objective is not to quantify before–after designation effects at the individual site level, but rather to assess long-term environmental trajectories within areas that currently constitute the Natura 2000 network, and to compare these trajectories with national background trends. It is acknowledged that Natura 2000 sites differ systematically from the national landscape in terms of land-cover composition, as they are preferentially designated in semi-natural and natural ecosystems. Consequently, the comparison presented here is not intended to isolate the causal effect of legal protection per se. Instead, it provides a contextualized assessment of whether areas currently forming the Natura 2000 network exhibit distinct long-term environmental trajectories relative to the broader landscape under shared climatic forcing. This approach is commonly applied in large-scale remote sensing assessments of protected area effectiveness, where cumulative landscape-level patterns and relative differences in long-term dynamics are emphasized over site-specific causal attribution.

### Data acquisition and pre-processing

Data were obtained from the Landsat 7 ETM+ Level-2 Tier-1 surface reflectance collection (landsat/LE07/C02/T1_L2), available via Google Earth Engine (GEE). This single-sensor dataset ensures consistent spectral resolution across the entire study period (2004–2023), thereby avoiding cross-sensor calibration issues^21^.

To mitigate the known Scan Line Corrector (SLC-off) malfunction affecting Landsat 7 data since May 2003, we created seasonal median composites (April–September) using at least eight valid scenes per year. This method reduced striping-related data loss to below 2% of the composite area, a level considered acceptable in regional remote sensing applications^22,23^.

Atmospheric correction, surface reflectance calculation, and cloud and shadow masking were applied using the QA_PIXEL quality band, following USGS Collection 2 processing guidelines (USGS, 2021).

To eliminate spectral distortion caused by water bodies—particularly for NDVI and TC Wetness—we masked permanent open water using the modified NDWI (mNDWI) with a threshold of > 0.3^24^. This was especially important for large aquatic SACs, such as “Zatoka Pucka i Półwysep Helski,” where over 90% of the site area consists of open water.

All indicators were computed for each SAC site and for the Polish territory as a whole, using the mean pixel values within site boundaries for each seasonal composite. For the national-scale background, indicators were calculated using the same processing workflow, seasonal composites, and spatial resolution to ensure methodological consistency rather than land-cover equivalence.

Landsat 7 SLC-off data, despite gap-filling procedures and seasonal compositing, may still introduce minor spatial artefacts. In addition, the spectral indices used in this study represent indirect, surface-level proxies; therefore, trends observed in the satellite record cannot be interpreted as direct measures of changes in ecological condition, habitat quality, or biodiversity without complementary field-based information.

Some Natura 2000 Special Areas of Conservation include habitat types with naturally sparse vegetation or exposed substrates (e.g., dry grasslands, dunes, rocky habitats), which may exhibit spectral responses distinct from forested or wetland ecosystems. To limit their potential influence on aggregated indicators, the analysis was conducted using site-level mean values derived from seasonal composites and restricted to sites exceeding 250 ha. This approach reduces the impact of localized spectral extremes and supports robust national-scale trend assessment.

### Spectral indices

To evaluate long-term ecological dynamics, we selected four well-established remote sensing indices representing both structural and functional properties of ecosystems.

NDVI (Normalized Difference Vegetation Index) is a widely used indicator of vegetation greenness, calculated as:

$$NDVI=\frac{{\rho NIR - \rho Red}}{{\rho NIR~+~\rho Red}}$$

where ρNIR and ρRed represent reflectance in the near-infrared and red bands, respectively. NDVI values range from − 1 to + 1, with higher values indicating greater vegetation greenness and fractional cover (e.g.,^25,15^.

TC Wetness is the moisture-related component of the Tasseled Cap transformation, calculated using coefficients adapted for Landsat 7^26^:

$$WET~=0.2626*\rho Blue+0.2141~*~\rho Green+0.0926*\rho Red$$

$$+0.0656*\rho NIR - 0.7629*\rho SWIR1 - 0.5388~*~B7$$

where: ρBlue, ρGreen, ρRed, ρNIR, ρSWIR1 and ρSWIR2 denote surface reflectance in the respective spectral bands.

TC Wetness captures relative variation in moisture-related spectral properties of vegetation and soil surfaces and is therefore used as a moisture-sensitive complement to NDVI.

NDBSI (Normalized Difference Built-up and Soil Index) is a composite spectral indicator representing the presence of bare soil and impervious surfaces. It is computed as the average of the Soil Index (SI) and Index-Based Built-up Index (IBI), following Xu^27^ and Liu et al.,^28^:

$$NDBSI=\frac{{SI+IBI}}{2}$$

In predominantly natural and semi-natural landscapes, such as Natura 2000 Special Areas of Conservation, NDBSI values primarily reflect variation in surface exposure and dryness associated with bare soil, sparse vegetation, or sealed surfaces, rather than systematic changes in urban land cover. Higher NDBSI values therefore indicate a greater proportion of exposed or sealed surfaces, commonly associated with lower habitat quality.

LST (Land Surface Temperature) represents the thermal radiance emitted from the land surface. It was estimated from the Landsat 7 thermal band using a standard radiometric conversion (EROS Center, 1999):

$$LST=B6*0.00341802+149.0~~$$

where:

B6 - denotes the digital number (DN) of the thermal band.

LST characterizes spatial and temporal variation in land surface, thermal conditions associated with land cover, vegetation presence, and surface properties.

While the indices used in this study are robust and widely applied, they represent indirect proxies of surface environmental conditions rather than direct measures of ecological processes or biodiversity, and may be influenced by seasonal variability, atmospheric conditions, and land cover heterogeneity^14^.

### Statistical analyses

For each of the four indicators, a 20-year time series (2004–2023) was constructed for two spatial domains: Natura 2000 Special Areas of Conservation, aggregated at the network level, and the national territory excluding Natura 2000 areas. To characterize long-term temporal trajectories and rates of change, we applied two robust non-parametric trend analyses:

- Mann–Kendall trend test – to detect the presence and direction of monotonic temporal trends, using Kendall’s tau (τ) and significance threshold α = 0.05;
- Sen’s slope estimator – to quantify the rate of change in indicator values over time, based on the median of all pairwise slopes^29^.

Both methods are resilient to outliers, non-normal distributions, and heteroscedasticity, making them suitable for ecological time series^30^. Before applying the trend tests, all-time series were screened for first-order autocorrelation using the lag-1 autocorrelation coefficient (ρ). Since |ρ| < 0.1 in all cases, pre-whitening correction was deemed unnecessary.

To facilitate relative comparison of long-term dynamics between the Natura 2000 network and the national background, we calculated:

- Δ slope – the difference in Sen’s slope estimates between Natura 2000 and the national background (Natura 2000 minus national background),
- Slope ratio **–** the ratio of Sen’s slope estimates, expressing the relative rate of change in Natura 2000 compared to the national background.

These comparative metrics are intended to describe relative differences in the pace and direction of long-term environmental change, rather than to infer causal effects of legal protection. All analyses were conducted in R (version 4.3.2) using the packages: Kendall, trend, zyp, dplyr, ggplot2, and data.table.

### Quasi-experimental robustness test using matched ring controls

To address potential selection bias, we implemented a paired ring-control robustness test using site-specific “ring” control areas located in the immediate surroundings of each Natura 2000 site (paired spatial counterfactual). Each protected site was paired only with its own adjacent ring control, which preserves local environmental context (climate zone, regional land-use pressure) and avoids comparing sites across different parts of Poland.

Importantly, this procedure is a *paired counterfactual design* rather than a statistical matching algorithm: we did not reweight, prune, or replace pairs to force baseline equivalence. Instead, we *assessed* baseline comparability over 2004–2006 by comparing mean baseline levels between Natura 2000 and ring controls using standardized mean differences (SMD) and non-parametric tests, and we then evaluated effectiveness primarily through *differences in trend estimates* (ΔSen = Sen_N2000 − Sen_Control) over 2004–2023. This trend-based comparison reduces sensitivity to baseline level differences because it tests whether trajectories diverge between paired protected and adjacent non-protected landscapes.

## Results

### Vegetation dynamics (NDVI)

**Fig. 2:**
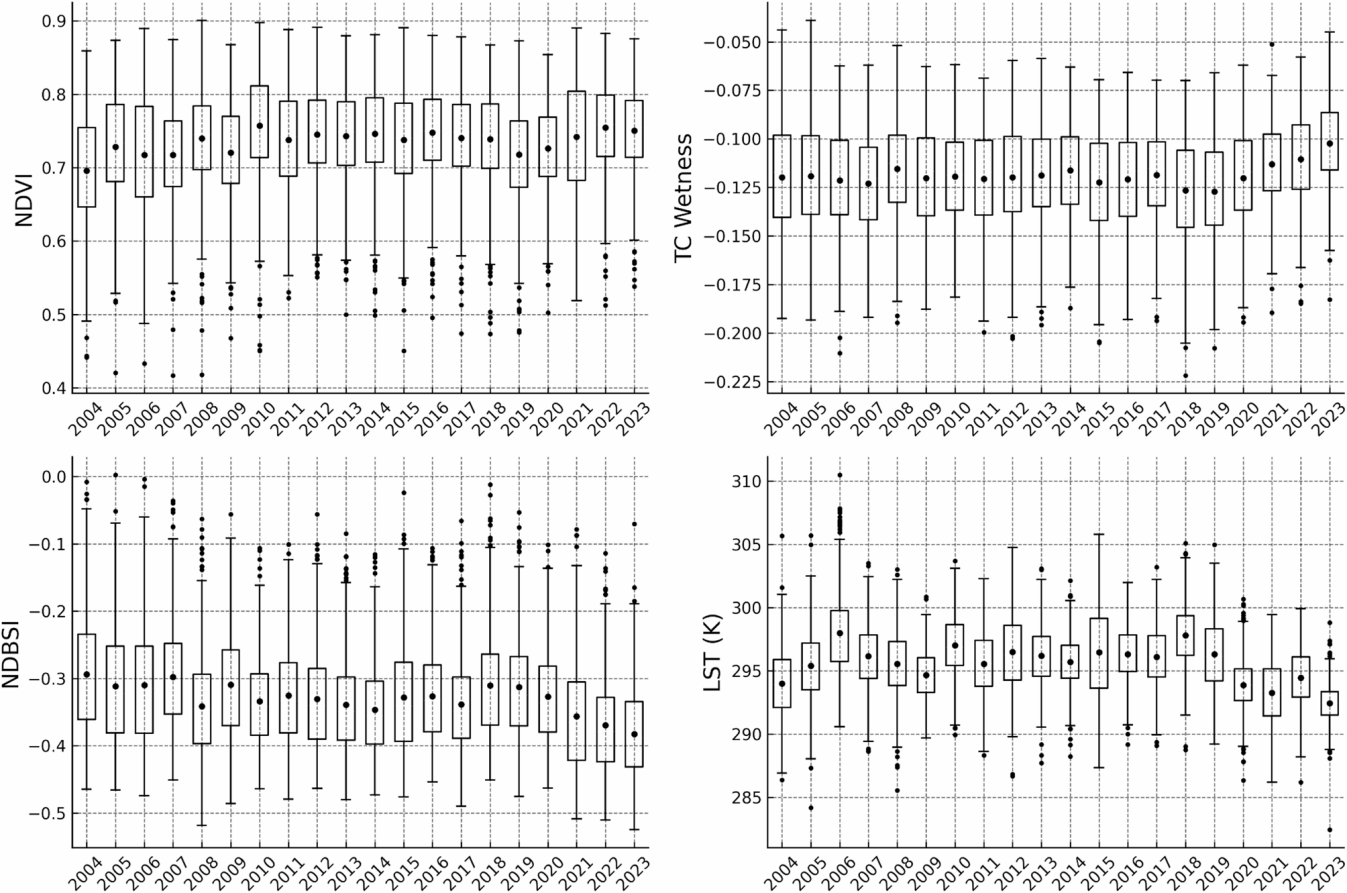
Temporal variation of selected remote sensing indicators within Natura 2000 sites in Poland during the period 2004–2023. Boxplots represent annual distributions of site-level mean values of NDVI, Tasseled Cap Wetness (TC Wetness), the Normalized Difference Built-up and Soil Index (NDBSI), and Land Surface Temperature (LST, in Kelvin) calculated for the April–September vegetation season.

Median NDVI values within Natura 2000 sites remained consistently high over the 20-year study period, ranging from 0.6978 in 2004 to 0.7542 in 2023 (Fig. 2). The standard deviation across years was low (0.0162), indicating minimal interannual variation. The distribution of NDVI values became increasingly compact over time, with narrower interquartile ranges and outliers occurring exclusively on the lower end (*n* = 112), reflecting increasing homogeneity in NDVI-derived vegetation greenness across protected areas.

The highest median value was recorded in 2010 (0.7663), with no years showing substantial downward shifts. Median NDVI remained high throughout the period, indicating consistently high NDVI values across the Natura 2000 network.

**Fig. 3:**
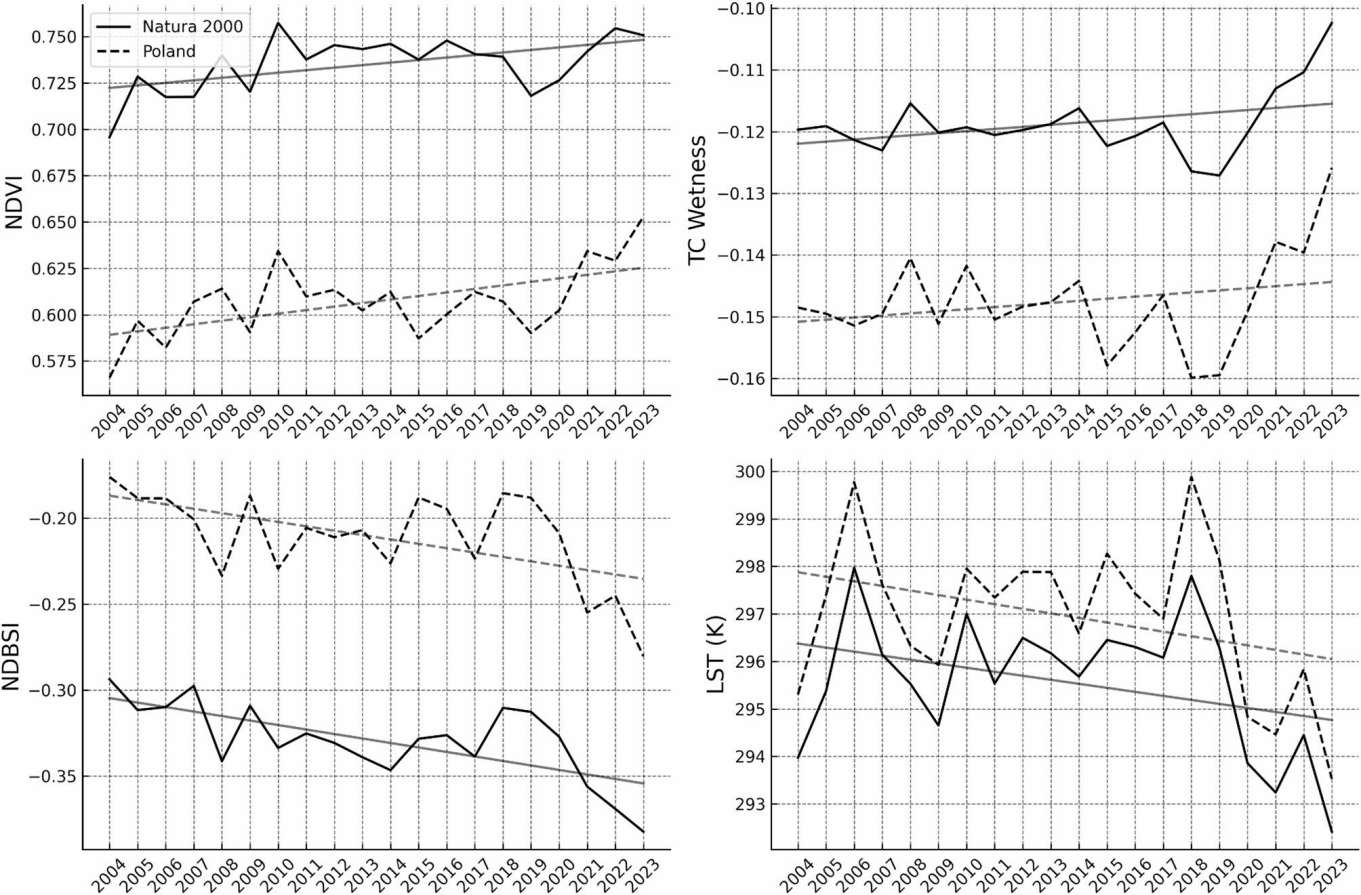
Long-term trajectories of four environmental indicators—NDVI, Tasseled Cap Wetness (TC Wetness), the Normalized Difference Built-up and Soil Index (NDBSI), and Land Surface Temperature (LST)—for Natura 2000 sites and the national background of Poland over the period 2004–2023. Lines represent annual mean values aggregated at the site level for Natura 2000 areas and at the national scale for Poland.

Statistical trend analysis supports this descriptive pattern: both protected and unprotected areas exhibited significant monotonic increases in NDVI (τ_N2000 = 0.368, *p* = 0.024; τ_PL = 0.326, *p* = 0.047), though the rate of increase was higher at the national level (Sen slope = 0.001818) than within Natura 2000 (0.001099) (Table 1). The slope ratio of 0.60 indicates that NDVI increased in protected areas at approximately 60% of the national rate. At the same time, the slightly higher Kendall’s τ value for Natura 2000 compared with the national background suggests a more consistent monotonic direction of change in the NDVI trend over time, without implying differences in vegetation quality or ecological condition (Fig. 3).

### Surface moisture conditions (TC Wetness)

TC Wetness values within Natura 2000 sites showed a slight upward shift over time, changing from − 0.1172 in 2004 to − 0.1002 in 2023 (Fig. 2). This 0.017-point shift was modest but steady, with the highest value recorded in 2023 and the lowest in 2019. The standard deviation across years was 0.0049—the lowest among all four indicators— indicating low interannual variability in TC Wetness values.

Outliers (*n* = 27) were almost entirely located on the lower end, with lower-than-typical values appearing in several individual years. Over time, the interquartile range narrowed and the distribution became more centralized, reflecting increasing uniformity in TC Wetness values across Natura 2000 sites.

Statistical trend analysis indicated no significant monotonic trends in TC Wetness for either Natura 2000 sites or the national background (τ_N2000 = 0.158, *p* = 0.351; τ_PL = 0.137, *p* = 0.422), and Sen slope estimates were similarly low (0.000179 and 0.000199, respectively) (Table 1). The near-identical slope ratio (0.90) indicates that both domains exhibited similarly low rates of change, while the slightly higher Kendall’s τ value for Natura 2000 reflects greater consistency in the direction of TC Wetness variation over time, without implying systematic differences in hydrological conditions (Fig. 3).

### Non-vegetated surface exposure (NDBSI)

Unlike NDVI and TC Wetness, the NDBSI exhibited a strong and consistent downward trend in both spatial domains, indicating a consistent decrease in NDBSI values over time. In Natura 2000 sites, the median NDBSI dropped from − 0.2982 in 2004 to − 0.3881 in 2023, with a relatively high standard deviation of 0.0236 (Fig. 2). Outliers (*n* = 110) occurred only on the upper end, suggesting occasional episodes of higher surface exposure or spectral dryness, but no extreme decreases.

Interquartile ranges contracted over time, and distributions became more centralized around lower NDBSI values. These patterns indicate a uniform and persistent shift toward lower NDBSI values across the Natura 2000 network.

Statistically, both domains showed significant negative trends (τ_N2000 = − 0.453, *p* = 0.005; τ_PL = − 0.337, *p* = 0.040). Sen slopes were − 0.002823 in Natura 2000 sites and − 0.002641 in the national background, yielding a slope ratio of 1.0689 and a slope difference of − 0.000182 (Table 1). These values indicate that the decline in NDBSI was slightly stronger in protected areas, corresponding to an approximately 7% faster rate of decrease in NDBSI values (Fig. 3), and the trend exhibited greater directional consistency in Natura 2000 sites.

**Table 1:** Summary of temporal trend analysis for four remote sensing indicators (NDVI, TCW, NDBSI, and LST) calculated for Natura 2000 areas (N2000) and the national background of Poland (PL) using the Mann–Kendall test and Sen’s slope estimator over the period 2004–2023.

Index	τ N2000	*p* N2000	Sen slope N2000	τ PL	*p* PL	Sen slope PL	Δ slope (N2000 - PL)	Ratio slope (N2000/PL)
NDVI	0,368	0,024	0,001	0,326	0,047	0,002	-0,001	0,605
TCW	0,158	0,351	0,000	0,137	0,422	0,000	0,000	0,899
NDBSI	-0,453	0,005	-0,003	-0,337	0,040	-0,003	0,000	1,069
LST	-0,147	0,386	-0,075	-0,168	0,319	-0,091	0,016	0,825

### Land surface temperature (LST)

LST exhibited the highest interannual variability among the indicators, with median values ranging from 292.45 K (2023) to 297.64 K (2018), and a standard deviation of 1.34 K (Fig. 2). Outliers occurred on both ends (high: *n* = 52; low: *n* = 57), suggesting persistent heterogeneity in land surface temperature patterns across SACs.

Although the years 2015–2019 recorded the highest median values, the final three years of the series (2021–2023) consistently showed lower surface temperatures. Nonetheless, the interquartile range remained wide throughout the study period, indicating substantial spatial variability and complex spatial and temporal variation not easily captured by monotonic trend tests.

Statistical analysis did not confirm significant monotonic trends in either domain (τ_N2000 = − 0.147, *p* = 0.386; τ_PL = − 0.168, *p* = 0.319). Sen slopes estimates were − 0.074743 for Natura 2000 sites and − 0.090620 for the national background (Table 1). The slope ratio was 0.8248, indicating a lower absolute magnitude of the estimated temperature change. Outliers were symmetrically distributed, and the Mann–Kendall test detected no monotonic trend in either domain, underscoring the dominance of interannual and spatial variability over long-term directional change.

### Comparative synthesis

When aggregated across all indicators, Natura 2000 sites were characterized by higher NDVI values, slightly stronger negative trends in NDBSI, and consistently lower LST values relative to the national background. NDVI increased at a slower rate within protected areas, whereas NDBSI decreased at a slightly faster rate in terms of indicator values. TC Wetness exhibited low-magnitude and statistically non-significant trends in both domains. LST trends were not statistically significant in either domain, although median LST values remained consistently lower within SACs across the study period.

These quantitative differences are summarized in Table 2, which presents Sen slopes estimates, slope differences, slope ratios, and Kendall’s τ for all indicators.

**Table 2:** Comparative trends in remote sensing–based indicators (2004–2023) within Natura 2000 sites and the national background of Poland, expressed as Sen’s slope estimates, slope differences, slope ratios, and Kendall’s τ.

Indicator	Sen slope (N2000)	Sen slope (Poland)	Δ slope (N2000 - PL)	Slope ratio (N2000 / PL)	Kendall’s τ (N2000 / PL)
NDVI	0,001099	0,001818	-0,000719	0,6	0.368 / 0.326
TC Wetness	0,000179	0,000199	-0,00002	0,8989	0.158 / 0.137
NDBSI	-0,002823	-0,002641	-0,000182	1,0689	-0.453 / -0.337
LST	-0,074743	-0,09062	0,015878	0,8248	-0.147 / -0.168

### Robustness analysis using paired ring controls

Baseline comparison revealed substantial initial differences between Natura 2000 sites and their surrounding ring controls (2004–2006). NDVI and NDBSI exhibited large standardized mean differences (SMD = 1.59 and − 2.48, respectively), confirming non-random site selection and distinct baseline biophysical characteristics. Wetness also showed considerable imbalance (SMD = 0.96), whereas LST exhibited relatively small baseline difference (SMD = − 0.07). Despite these baseline disparities, paired trend comparison (2004–2023) revealed consistent patterns. For NDVI, the median paired difference in Sen’s slope was − 0.00031 (*p* = 7.18 × 10⁻⁶), indicating slightly slower vegetation greening inside Natura 2000 compared to adjacent controls. However, the magnitude of the effect was small, and 39.6% of sites exhibited positive ΔSen. For NDBSI, the median ΔSen was − 0.000587 (*p* = 1.13 × 10⁻¹⁶), suggesting significantly stronger soil exposure and degradation trends in control areas relative to protected sites. Only 32.1% of sites showed positive ΔSen, indicating that Natura 2000 areas were less affected by increasing bare soil or built-up signatures. For Wetness, the median ΔSen was − 0.000111 (*p* = 9.70 × 10⁻⁶), reflecting marginally slower wetness increases within protected areas compared to controls. For LST, no significant paired difference was observed (median ΔSen = 0.000804; *p* = 0.84), suggesting similar warming trajectories inside and outside Natura 2000 sites.

Overall, the robustness test confirms that the most pronounced divergence between protected and control areas is observed for NDBSI, while NDVI and Wetness effects are statistically significant but small in magnitude, and LST shows no differential trend. These paired comparisons isolate local counterfactual differences relative to immediately adjacent landscapes and therefore provide a more conservative test of differential temporal trajectories than national-scale contrasts presented in earlier sections.

## Discussion

The paired ring-control robustness analysis provides a conservative quasi-experimental framework for interpreting the national-scale contrasts presented in the Results section. While substantial baseline imbalance confirms non-random site selection, trend-based ΔSen comparisons allow evaluation of whether protected and adjacent non-protected landscapes diverge over time. The robustness results indicate that the clearest differential trajectory is observed for NDBSI, whereas NDVI and TC Wetness effects are statistically detectable but small in magnitude, and LST shows no significant divergence. The following sections interpret each indicator in light of both national-scale contrasts and paired counterfactual comparisons.

### Vegetation greenness trajectories and landscape stability

The results indicate that vegetation greenness in Poland, as reflected by NDVI, increased moderately over the past two decades. This pattern is consistent with the broadly documented “greening” signal observed across Central and Eastern Europe, largely attributed to climate-driven lengthening of the growing season, CO₂ fertilization, and gradual land-use change^18,31,32^. However, when compared to the national background, the pace of NDVI growth was approximately 40% lower within Natura 2000 Special Areas of Conservation according to Sen’s slope estimates. Importantly, the paired ring-control robustness test indicates that this divergence is statistically detectable but small in magnitude at the local scale (median ΔSen = − 0.00031), suggesting that the difference reflects subtle moderation rather than strong buffering of greening dynamics. This slower trajectory is consistent with relatively stable greenness dynamics in areas that already exhibit high baseline NDVI values.

Comparable patterns have been reported elsewhere in Europe: protected landscapes often display lower rates of NDVI increase but higher long-term stability^16,33^. In Poland, the pattern likely reflects both pre-existing ecological value (sites selected for protection already represented habitats with high baseline vegetation greenness) and the continuation of low-intensity land use following designation (Grodzińska-Jurczak & Cent, 2011). Importantly, Natura 2000 sites in Poland were designated over an extended period rather than at a single point in time. As a result, the temporal trends identified in this study do not represent uniform post-designation responses across all sites. For areas designated later, early segments of the analyzed time series may reflect pre-protection conditions. Therefore, the observed NDVI trajectories should be interpreted as long-term environmental dynamics within areas that currently form the Natura 2000 network, rather than as direct before–after effects of legal protection. Such landscapes may exhibit limited capacity for further increases in NDVI compared to more intensively transformed or recently abandoned areas.

The divergence between SACs and the surrounding landscape is consistent with a buffering pattern, whereby areas with high baseline vegetation greenness exhibit slower and more internally consistent temporal change. Previous studies have shown that landscapes characterized by high vegetation cover and limited land-use intensity can exhibit reduced sensitivity to large-scale environmental fluctuations^16^.

The narrow interquartile ranges and low NDVI variability observed here strengthen this interpretation.

Overall, the results indicate that areas currently designated as Natura 2000 sites are characterized by more stable long-term NDVI trajectories than the surrounding national landscape. This stability is expressed not through accelerated greenness increases, but through higher baseline values, slower rates of change, and greater directional consistency over time. These patterns should be interpreted as descriptive differences in long-term environmental dynamics associated with protected areas, rather than as direct evidence of causal effects of legal protection. The robustness analysis confirms that Natura 2000 sites were not biophysically comparable to adjacent landscapes at baseline (SMD_NDVI = 1.59), reinforcing that the observed divergence in greenness trajectories reflects differences in long-term landscape context rather than post-designation treatment effects. The paired ΔSen estimate (− 0.00031; *p* = 7.18 × 10⁻⁶) indicates a statistically detectable but small moderation of greening dynamics at the local scale. From a national-scale perspective, this contributes to ongoing discussions on how protected area networks relate to landscape-level stability under shared climatic and land-use pressures.

### Moisture-related surface dynamics under dry conditions

TC Wetness trends remained broadly stable across the 20-year study period, with no statistically significant increase or decrease. At first glance this may appear unremarkable; however, it contrasts with regional projections that anticipate increasing moisture deficits in Central Europe under future climate scenarios^34^; European Environment Agency, 2021; 2023).

The absence of a clear drying trend should not be interpreted as trivial. During extreme dry years—2003, 2015, and 2018, among the most severe droughts in Europe since 1950^35^—clear negative anomalies were recorded in TC Wetness values. This confirms that the index is sensitive to hydrometeorological extremes and capable of capturing short-term deviations in moisture-related spectral properties.

During these dry years, SACs exhibited smaller declines in TC Wetness values and narrower interannual variability compared to the national background. This pattern is consistent with differences in land-cover composition between protected and non-protected landscapes.

Natura 2000 sites in Poland include a higher proportion of wetlands, peatlands, riparian zones, and forested areas, which are associated with more stable moisture-related spectral responses than intensively managed or artificially drained landscapes^19,^^36^. In addition, the lower intensity of land-use modification within many SACs may contribute to reduced variability in surface moisture-related indices.

While stable long-term trends may superficially suggest limited change, in the context of comparative landscape analysis they indicate relative persistence of moisture-related surface conditions. Several studies emphasize that maintaining long-term stability in moisture-sensitive surface indicators is relevant for understanding how landscapes respond to increasing climatic variability^34^; European Environment Agency, 2021). Even if episodic droughts influence species composition or regeneration processes, such effects cannot be directly inferred from TC Wetness alone.

Taken together, these results suggest that, beyond vegetation greenness, Natura 2000 sites are characterized by more stable moisture-related surface dynamics at the landscape scale compared to the national background. The paired ring-control comparison indicates that the difference in TC Wetness trends relative to adjacent landscapes is statistically significant but small (median ΔSen = − 0.000111; *p* = 9.70 × 10⁻⁶), suggesting modest moderation rather than strong hydrological buffering. These patterns should be interpreted as descriptive contrasts rather than causal effects of legal protection.

### Land surface exposure dynamics (NDBSI trends)

The NDBSI results reveal a systematic decline in the extent of exposed and impervious surfaces in Poland between 2004 and 2023. This downward trajectory is observed both nationally and within Natura 2000 sites, but the decrease was slightly stronger inside the protected network (Sen’s slope ≈ − 0.00282 vs. − 0.00264 nationally). Although modest in absolute terms, this difference indicates a slightly stronger decrease in NDBSI values within areas currently designated as Natura 2000 sites.

Higher NDBSI values typically represent exposed soil surfaces, impervious areas, or degraded vegetation cover. A negative NDBSI trend indicates a reduction in surface exposure as captured by the spectral index, which may be associated with a variety of land-cover dynamics in predominantly non-urban landscapes. In Poland, such patterns are consistent with documented land-use changes, including agricultural abandonment and reduced intensity of surface modification in some regions^28,27^. Within Natura 2000 sites, land-use regulations are associated with lower rates of surface modification compared to more intensively transformed areas (Grodzińska-Jurczak & Cent, 2011).

Protected areas particularly benefit from regulatory barriers against construction in wetlands, forests, and dunes, which are associated with vegetation-dominated surface characteristics under low disturbance.

Although certain forms of land conversion still occur within SACs, including residential development or tourism infrastructure, these pressures are spatially limited^20,19^.

The NDBSI results indicate that areas currently designated as Natura 2000 sites exhibit slightly stronger long-term decreases in surface exposure compared to the national background. Importantly, the paired ring-control robustness test reinforces this pattern: the median paired difference in Sen’s slope was − 0.000587 (*p* = 1.13 × 10⁻¹⁶), with only 32.1% of sites exhibiting positive ΔSen. Despite substantial baseline imbalance (SMD = − 2.48), the directional consistency of the paired comparison suggests that Natura 2000 areas experienced relatively weaker soil exposure and surface degradation dynamics compared to immediately adjacent landscapes. These differences should be interpreted as conservative quasi-experimental contrasts rather than direct causal evidence of land recovery.

### Surface thermal dynamics (LST trends)

Despite the well-documented regional warming trend in Poland, no statistically significant increase in Land Surface Temperature (LST) was detected in either Natura 2000 sites or the national background during 2004–2023. The Sen slopes were weakly negative (≈ − 0.07 K/year in SACs vs. − 0.09 K/year nationally), and neither reached statistical significance (τ_N2000 = − 0.147, *p* = 0.386; τ_PL = − 0.168, *p* = 0.319; see Table 1).

This finding may appear counterintuitive given the continuous increase in air temperatures recorded by meteorological stations across Central Europe^34^; EEA, 2021). However, several factors may contribute to this discrepancy. First, LST reflects surface–atmosphere interactions rather than free-air conditions, and it is strongly mediated by land cover type and vegetation density, with forests consistently associated with lower LST values^37,38^. Second, interannual variability was substantial: median LST ranged from 292.45 K (2023) to 297.64 K (2018), with a standard deviation of ~ 1.34 K. The warm extremes of 2015–2019 contrast with a cluster of cooler years in 2021–2023, producing no monotonic trend.

Importantly, SACs consistently exhibited lower LST values than the national background. This pattern is consistent with differences in land-cover composition, as areas dominated by forests, wetlands, and semi-natural grasslands are commonly associated with lower surface temperatures in satellite-based studies^14,39^. Such contrasts reflect well-established relationships between land cover and surface thermal properties rather than direct causal effects of legal protection.

Evidence from Poland corroborates this pattern. During the record-breaking summer of 2018, surface temperatures in forested Natura 2000 sites were several degrees cooler than in adjacent agricultural areas^40^. Such localized contrasts highlight the capacity of different land-cover types to modulate surface thermal conditions, even in the absence of significant long-term trends.

The slight downward slope observed in both protected and unprotected areas may partly reflect broader land-cover dynamics occurring over the study period, although these relationships were not explicitly tested here. Thus, while air temperatures rise, surface thermal environments are modulated by land-use dynamics.

In sum, although LST trends were not statistically significant, areas currently designated as Natura 2000 sites consistently exhibited lower surface temperatures than the national background. These differences should be interpreted as descriptive contrasts in surface thermal conditions associated with land-cover composition, rather than as evidence of causal cooling effects or climate buffering. Consistent with this interpretation, the paired ring-control comparison detected no significant difference in warming trajectories between Natura 2000 sites and adjacent landscapes (median ΔSen = 0.000804; *p* = 0.84), confirming the absence of differential thermal buffering at the local scale.

### Conservation policy implications and network effectiveness

The results provide several insights into the functioning of Natura 2000 as a large-scale conservation framework when viewed through the lens of long-term environmental indicators. First, the relatively stable trajectories observed across multiple satellite-derived indices suggest that areas currently designated as Special Areas of Conservation are characterized by lower rates of structural land surface change (particularly NDBSI) and slightly moderated greenness and moisture dynamics compared to the national background. The paired ring-control robustness analysis indicates that this pattern is most consistently supported for land surface exposure dynamics (NDBSI), while differences in greenness and moisture trajectories are statistically significant but modest in magnitude, and no differential thermal trend was detected. This pattern is consistent with previous European studies reporting that protected landscapes often exhibit slower land-use dynamics and more persistent surface characteristics over time^3,16^.

Second, the observed contrasts between SACs and the national background highlight differences associated with legal designation and land-cover composition. While broader greening trends and land-cover changes at the national scale may reflect socio-economic processes such as agricultural abandonment, the consistently lower LST values, reduced NDBSI trends, and more stable TC Wetness trajectories observed within SACs indicate that protected areas follow distinct long-term environmental pathways relative to the surrounding landscape^4,41^.These differences should be interpreted as descriptive patterns rather than direct evidence of causal effects of conservation measures.

Third, the study underscores the value of satellite-based monitoring for conservation policy and reporting. Field-based ecological surveys remain essential but are often limited in spatial and temporal coverage. In contrast, long-term remote sensing data provide a consistent and scalable means of tracking environmental trajectories across extensive protected area networks, complementing site-level assessments and supporting periodic reporting requirements under the EU Habitats Directive^42,17^.

Finally, from a policy perspective, the results suggest that Natura 2000 sites represent areas with relatively stable long-term environmental characteristics under shared climatic and land-use pressures. These characteristics reflect both pre-existing environmental conditions—since many sites were designated due to their high natural value—and the continuation of land-use regimes that limit intensive surface modification. Rather than demonstrating effectiveness in a causal sense, the findings provide a landscape-scale context for understanding how protected area networks are associated with persistent environmental patterns relevant to biodiversity conservation and land-use planning within broader European sustainability frameworks. While causal inference is beyond the scope of this study, the use of multiple complementary indicators provides a consistent descriptive basis for comparing long-term surface-level patterns across landscapes. The incorporation of paired spatial counterfactual comparisons strengthens internal validity relative to purely national-scale contrasts, while acknowledging the inherent baseline differences between protected and non-protected landscapes.

### Limitations

This study relies exclusively on satellite-derived indicators, which capture structural and surface-level properties of ecosystems but do not directly measure species-level biodiversity or functional ecological processes. The moderate spatial resolution of Landsat imagery (30 m) may obscure fine-scale habitat changes, particularly in heterogeneous SACs. LST estimation from a single thermal band is sensitive to emissivity assumptions and may not fully reflect near-surface air temperature dynamics. In addition, the Mann–Kendall test identifies monotonic trends only and cannot detect non-linear or abrupt ecological transitions.

Furthermore, Natura 2000 Special Areas of Conservation in Poland were designated over an extended period rather than simultaneously. As a result, the long-term trends observed in this study reflect cumulative environmental trajectories within areas that currently form the Natura 2000 network, rather than direct before–after effects of legal protection. Consequently, observed differences may partly reflect pre-existing environmental conditions or land-use characteristics, and causal attribution of detected trends to the Natura 2000 designation should therefore be approached with caution. Although the paired ring-control design improves internal validity by comparing protected sites with their immediate surroundings, it does not fully eliminate structural baseline differences, particularly where Natura 2000 sites encompass habitat types absent from adjacent landscapes. Therefore, the robustness analysis should be interpreted as a conservative quasi-experimental comparison rather than a fully balanced causal design.

## Conclusions

This study presents the first national-scale, two-decade satellite-based assessment of environmental trajectories within Natura 2000 Special Areas of Conservation (SACs) in Poland, integrating indicators of vegetation greenness, moisture-related surface properties, land surface exposure, and land surface temperature. Across all indicators, areas currently designated as SACs consistently exhibited higher NDVI values, lower NDBSI values, and lower land surface temperatures compared to the national background. During drought years, SACs also showed smaller relative declines in TC Wetness values, indicating greater persistence of moisture-related spectral signals.

Taken together, the results indicate that areas forming the Natura 2000 network are characterized by more stable long-term surface-level environmental trajectories than the surrounding landscape over the period 2004–2023. These patterns reflect both pre-existing environmental characteristics of sites selected for protection and the continuation of land-use regimes associated with limited surface modification. Rather than demonstrating causal effects of legal protection, the observed differences describe how protected landscapes are associated with persistent environmental conditions under shared climatic and land-use pressures^13,43–50^.

The multi-indicator remote sensing framework applied here provides a reproducible and cost-effective approach for evaluating long-term environmental trajectories of protected area networks at national scales. Such approaches can complement field-based assessments by offering consistent, landscape-level context for conservation monitoring and policy evaluation.

## Supplementary Information

Below is the link to the electronic supplementary material.

Supplementary Material 1

## Data Availability

All datasets generated and analysed during the current study, including annual Landsat composite rasters, derived spectral indices (NDVI, TC Wetness, NDBSI, LST), and site-level time-series summaries for all 330 Natura 2000 SACs, are publicly available. Raw satellite data are accessible from the USGS Landsat Collection 2 repository ( [https://earthexplorer.usgs.gov](https:/earthexplorer.usgs.gov) ). Processed datasets and the full analysis workflow will be deposited in the Zenodo public repository. A DOI will be provided upon acceptance.
