# Supplementary material for: Long-term remote sensing assessment of Natura 2000 protected areas in Poland (2004–2023)
Source: Sci Rep. 2026 Mar 7;16:12448. doi: 10.1038/s41598-026-42863-8 (PMC13083888; doi:10.1038/s41598-026-42863-8)
Supplement: Supplementary file 1 — Supplementary Material 1 [file 41598_2026_42863_MOESM1_ESM.pdf]

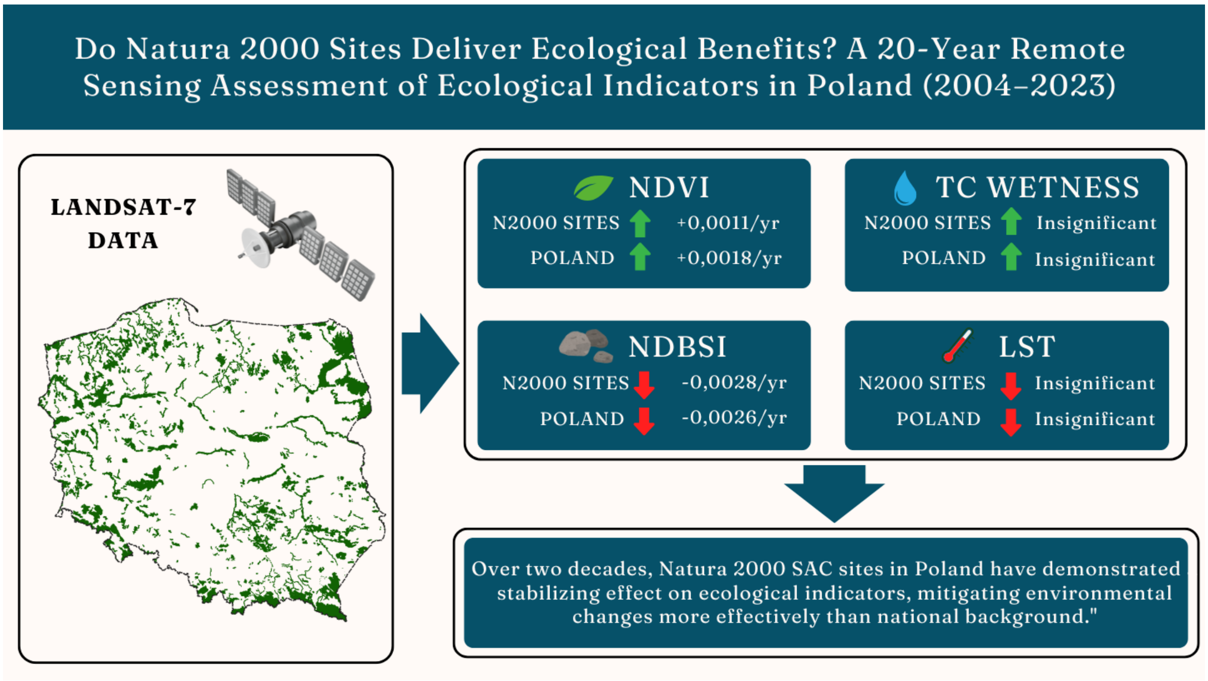
Do Natura 2000 Sites Deliver Ecological Benefits? A 20-Year Remote Sensing Assessment of Ecological Indicators in Poland (2004–2023)
LANDSAT-7 DATA
NDVI
N2000 SITES +0,0011/yr
POLAND +0,0018/yr
TC WETNESS
N2000 SITES Insignificant
POLAND Insignificant
NDBSI
N2000 SITES -0,0028/yr
POLAND -0,0026/yr
LST
N2000 SITES Insignificant
POLAND Insignificant
Over two decades, Natura 2000 SAC sites in Poland have demonstrated stabilizing effect on ecological indicators, mitigating environmental changes more effectively than national background."
